# Long-Acting Growth Hormone for Pediatric Growth Hormone Deficiency

**DOI:** 10.1210/jendso/bvaf040

**Published:** 2025-03-04

**Authors:** Norbert Albers, Sarah Cadarette, Ben Feakins, María Arregui, Stephen Ebohon, Pamela Lai, Subhara Raveendran, Mads Kjelgaard-Hansen, Christine Andersen, Carol Zhao, Alden Smith, Mitchell Geffner

**Affiliations:** Department of Pediatric Endocrinology, Christliches Kinderhospital, Osnabrueck 49074, Germany; Cencora, Conshohocken, PA 19428, USA; Cencora, Conshohocken, PA 19428, USA; Cencora, Hannover D-30159, Germany; Cencora, London WC2B 4PJ, UK; Ascendis Pharma Inc, Palo Alto, CA 94304, USA; Ascendis Pharma Inc, Palo Alto, CA 94304, USA; Ascendis Pharma A/S, Hellerup DK-2900, Denmark; Ascendis Pharma A/S, Hellerup DK-2900, Denmark; Ascendis Pharma Inc, Palo Alto, CA 94304, USA; Ascendis Pharma Inc, Palo Alto, CA 94304, USA; Center for Endocrinology, Diabetes, and Metabolism, The Saban Research Institute of Children's Hospital Los Angeles, Los Angeles, CA 90027, USA; Pediatrics, The Keck School of Medicine of USC, Los Angeles, CA 90089, USA

**Keywords:** pediatric growth hormone deficiency, long-acting growth hormone, meta-analysis, literature review

## Abstract

Long-acting growth hormone (LAGH) has the potential to improve adherence and outcomes over daily somatropin in growth hormone deficiency (GHD). Whereas daily somatropin products are molecularly identical, LAGHs are molecularly distinct; additional moieties or mechanisms that prolong LAGH action confer unique pharmacodynamic/pharmacokinetic properties that could affect efficacy and safety. Only one LAGH available in the United States and Europe (lonapegsomatropin) delivers unmodified somatropin. With no head-to-head clinical trials of LAGHs available, this systematic literature review and network meta-analysis were conducted to compare the relative efficacy and safety of LAGHs in pediatric GHD.

Five trials were eligible for inclusion in a Bayesian network meta-analysis; 3 contributed to the base case network, including 3 LAGHs (lonapegsomatropin, somapacitan, and somatrogon) and daily somatropin. Treatment with lonapegsomatropin was associated with statistically significantly higher annualized height velocity and change from baseline in height SD score (SDS) at week 52 compared to daily somatropin and somapacitan; no other significant differences in these outcomes were found. The change from baseline in average insulin-like growth factor-1 (IGF-1) SDS at week 52 was significantly higher for somatrogon vs all comparators and for lonapegsomatropin vs daily somatropin and somapacitan; average IGF-1 SDS was within normal range in all trials. No significant differences were seen in progression in bone age-to-chronological age ratio or serious adverse events (SAEs). Sensitivity analyses were consistent with the base case.

In this network meta-analysis, lonapegsomatropin was the only LAGH associated with better growth outcomes. No significant differences were detected regarding SAEs; other safety outcomes could not be analyzed.

Growth hormone (GH) is a polypeptide endocrine hormone produced in the pituitary gland that is involved in ensuring normal longitudinal height growth, muscle and bone strength, and body fat distribution in childhood and adolescence and maintaining metabolic health during adulthood [1, 2]; GH may also play a role in cognitive function [3]. The effects of GH are mediated through its actions in several types of tissues, including liver, cardiac muscle, adipose, cartilage, and skeletal muscle [4-8].

Pediatric growth hormone deficiency (pGHD) is a rare disorder in which inadequate secretion of GH by the anterior pituitary gland causes short stature and delayed skeletal maturation [9, 10]. The incidence of pGHD is approximately 2.15 (95% CI, 1.97-2.35) per 100 000 children, and the prevalence is estimated at approximately 1:4000 to 1:10 000 [11, 12]. While the symptoms of pGHD vary, in addition to poor growth, affected children may manifest low energy levels, poor sleep, decreased focus, and reduced muscle development [13]. Additional signs and symptoms include delayed dentition, delayed puberty, and mid-face hypoplasia [14]. If pGHD remains untreated, in addition to the physical effects this condition may also affect a child's social and emotional well-being, with effects attributed both to physical limitations (eg, reduced performance or exclusion from physical activity due to size) and social perceptions (eg, appearing younger than their age) [13, 15]. pGHD can either be congenital or acquired, have a known cause or be idiopathic, and either be isolated or occur in association with multiple other pituitary hormone deficiencies [16]. Specific causes of pGHD include pituitary tumors, genetic disorders, radiation, and optic nerve hypoplasia [17].

The current standard of care for the management of pGHD focuses on the initiation of treatment with GH replacement as soon as possible after diagnosis to achieve a normal height during childhood and when entering adulthood [9, 18]. While guidelines consider an increase in height to be the main goal of treatment, GH has additional metabolic effects, including improved body composition, through decreasing visceral fat mass and increasing lean body mass [19]. Treatment with GH is also associated with improved quality of life [20]. Currently available formulations of recombinant human growth hormone (rhGH) include once-daily subcutaneous injections or once-weekly subcutaneous long-acting growth hormone (LAGH) injections [18, 21].

While daily somatropin injections have long been a part of the standard of care within pGHD management, there is often a substantial treatment burden associated with daily administration, resulting in adherence challenges and, therefore, suboptimal growth outcomes [15, 21-23]. A study investigating the effect of daily somatropin injections on patients and caregivers found substantial burdens such as injection-site reactions, fear or unhappiness associated with injection administration, interference with daily life, and difficulty in accommodating injections into the daily schedule [15]. The effect of these burdens has been observed in multiple adherence studies. One such study assessing compliance with daily somatropin administration, defined as missing 1 or dose or less per week based on empty vials returned to the study center, found that 66% (73/110) of patients were noncompliant. Notably, better compliance was associated with faster height velocity SD score (SDS) [22]. Due to the challenges associated with daily somatropin injection therapy, LAGH may present a significant advantage through once-weekly or biweekly administration that has the potential to improve patient adherence and thus treatment outcomes [24, 25].

LAGH formulations consist of either native rhGH (somatropin) that is transiently modified or somatropin analogues that are permanently modified to extend their half-life [26]. The goal of achieving sustained exposure to GH led to the earlier development of depot and PEGylated forms of LAGH, and, more recently, to formulations based on noncovalent albumin-binding (somapacitan), fusion-protein (somatrogon), and prodrug (lonapegsomatropin) technologies [21]. Endogenous GH consists of 191 amino acids with a molecular weight of 22 kDa (kilodalton) [5]. Somapacitan consists of an hGH analogue with a variant in the GH amino-acid sequence permanently attached to a high-affinity albumin-binding moiety, with more than 99% of somapacitan in the circulation bound to plasma proteins [27, 28]. GH receptor (GHR) engagement still occurs while albumin-bound [29]. The combined weight of GH, the albumin binder, and endogenous albumin is 89.8 kDa [30]. Somatrogon consists of an hGH analogue fused to one copy of the C-terminal peptide from the β-chain of human chorionic gonadotropin at the N-terminus and 2 copies of the C-terminal peptide at the C-terminus with a molecular weight of the receptor-active moiety of approximately 40 kDa [31]. Unlike LAGH formulations containing permanently modified GH variants as described earlier, lonapegsomatropin and LB03002 (available only in South Korea) release and activate the GHR through unmodified somatropin. Lonapegsomatropin is a prodrug of somatropin with the identical 191 amino-acid sequence to endogenous GH conjugated to a carrier via a proprietary TransCon linker. The molecular weight of the receptor-active moiety is 22 kDa and is molecularly identical to somatropin [32-34]. LB03002 consists of rhGH encapsulated in sodium hyaluronate microparticles, with the molecular weight of the released rhGH being 22 kDa [35].

Whereas daily somatropin products are molecularly identical (somatropin), LAGHs are molecularly distinct, and the additional moieties or mechanisms of different LAGHs that prolong its action confer unique pharmacokinetic and pharmacodynamic properties that could affect efficacy and safety as a result of altered pharmacokinetic and tissue distribution [21]. To date, there have been no head-to-head trials comparing the efficacy of the different LAGH formulations in pGHD. Previous publications have synthesized the literature on LAGHs [36-39]. However, this analysis is unique in using a systematic literature review, using a Bayesian framework to conduct a network meta-analysis, and focusing on treatment with approved doses of LAGHs over 52 weeks. Thus, this systematic literature review and Bayesian network meta-analysis were conducted to compare the relative efficacy and safety of the approved LAGH formulations against each other and against daily somatropin in treatment-naive pGHD.

## Materials and Methods

### Systematic Literature Review

This systematic literature review adhered to the Preferred Reporting Items for Systematic Reviews and Meta-Analyses guidelines [40]. Searches were conducted in Embase and Medline (via Ovid) from database inception through August 10, 2023. Full search terms can be found in Supplementary Table S1 [41]. Conference abstracts indexed in Embase and published in or after 2021 were also included in the search, and abstracts from the 2 most recent annual meetings for the following were also screened: Endocrine Society, European Congress of Endocrinology, American Association of Clinical Endocrinology, and Pediatric Endocrine Society. Bibliographies of relevant systematic literature reviews and meta-analyses identified via the database searches published in or after 2019 were reviewed for additional citations.

Articles were included if they met the predefined criteria in Table 1. Titles and abstracts were independently screened by 2 researchers; any citations for which inclusion criteria were met were retrieved in full-text format. All full-text publications were independently screened by 2 researchers (S.C., B.F., M.A., and S.E. conducted screening of whom 2 were assigned to each publication). Any discrepancies between reviewers during the abstract or full-text screening were adjudicated by a third researcher. Data extraction was performed by one researcher and validation was conducted by a second researcher. Quality assessment of the included studies was carried out using the Cochrane Collaboration's tool for assessing the risk of bias in randomized controlled trials [42]. Due to limited information available in conference abstracts, quality assessment was confined to primary trials with full-text publications.

**Table 1.** Inclusion criteria for the systematic literature review

Domain	Inclusion criteria		
**Population**	Treatment-naive children (age ≤18 years) with pGHD		
**Interventions**	Any LAGH		
**Comparators**	Any treatment allowing for a connected network, including LAGH and daily somatropin		
**Outcomes*^a^***	Efficacy	Safety	
	Annualized height velocity	SAEs	Discontinuations due to AEs
	Height SDS	Total AEs	Injection-site reactions
	Bone age-to-chronological age ratio	Treatment-emergent AEs	Neutralizing antibodies
	IGF-1 SDS	Nonneutralizing anti-hGH antibodies	
	BMI	Dose reduction/temporary discontinuation due to AEs	
	BMI SDS		
**Study design**	Randomized controlled trials		
**Databases**	Embase, Medline, and select conference abstracts were searched through August 10, 2023		
**Language**	English-language publications		

Abbreviations: AE, adverse event; BMI, body mass index; hGH, human growth hormone; IGF-1, insulin-like growth factor-1; LAGH, long-acting growth hormone; pGHD, pediatric growth hormone deficiency; SAE, serious adverse event; SDS, SD score.

^a^Studies were eligible for inclusion regardless of how the outcome was reported (eg, as change from baseline, follow-up value, proportion of patients meeting a threshold).

### Feasibility Assessment for Network Meta-Analysis

A feasibility assessment was conducted to determine if the characteristics of the trials identified in the systematic literature review were similar enough to be quantitatively combined in a network meta-analysis [43]. This step minimizes potential biases arising from studies substantially differing in terms of characteristics that were effect modifiers. All studies were compared with respect to study design, patient and treatment characteristics, outcome definitions, and time points of evaluation. Additionally, potential threats to the validity of the analyses were assessed by evaluating clinical and methodological heterogeneity among studies. Studies contributing to significant heterogeneity were excluded from the base case analysis and were instead considered for inclusion in a sensitivity analysis. To be deemed feasible for meta-analysis, at least 2 studies within an analytic scenario were needed to report on the outcome of interest for approved doses of LAGH formulations at the 52-week time point in treatment-naive children with pGHD.

### Network Meta-Analysis

A Bayesian network meta-analysis was performed to obtain relative treatment effects for each analyzable outcome, using the generalized linear modeling framework recommended by the National Institute for Health and Care Excellence (NICE) Decision Support Unit [44]. An advantage of the Bayesian Markov chain Monte Carlo approach, compared to frequentist approaches, is that sampling from the posterior distribution simultaneously satisfies the requirements for statistical estimation and inference, while also serving as a platform for probabilistic decision-making [44]. Data reported at 52 weeks of treatment were used in the analysis. To maximize the extraction of relevant data, graphical digitization was used in certain instances to obtain data points.

Treatment effects were summarized by reporting the median of mean differences (continuous outcomes) or odds ratios (binary outcomes) along with corresponding 95% credible intervals (CrI), which encompass the range of values within which there is a 95% probability that the true parameter estimate lies. Additional detail on data extraction and analysis methods can be found in the supplementary network meta-analysis methods [41].

To estimate the average probability that a treatment was the best treatment for a specific outcome, surface under the cumulative ranking curve scores were calculated in each analysis. Surface under the cumulative ranking curve scores are a numerical representation of the overall ranking of treatments included in a network meta-analysis. The ability to calculate surface under the cumulative ranking curve scores is unique to using a Bayesian framework for network meta-analysis. Surface under the cumulative ranking curve rankings can be thought of as metrics to determine which treatment has the highest likelihood of achieving the best outcome [45]. The surface under the cumulative ranking curve score comprises a single value (0%-100%) for each treatment; values close to 100% indicate a high likelihood that the treatment is in one of the top ranks, while values close to 0% indicate that a treatment is ranked at or near the bottom. Surface under the cumulative ranking curve scores also provide a means for treatment ranking, indicating which treatment is most likely to be top-ranked for a given outcome. It should be noted that the surface under the cumulative ranking curve rankings may be heavily influenced by inclusions or exclusions from the corresponding network meta-analysis. Nevertheless, an aggregate evaluation of the surface under the cumulative ranking curve statistic, treatment rankings, and visual representations of relative effects compared to a reference treatment facilitates a comprehensive interpretation.

## Results

### Study Selection in the Systematic Literature Review and Feasibility Assessment

**Figure 1.**
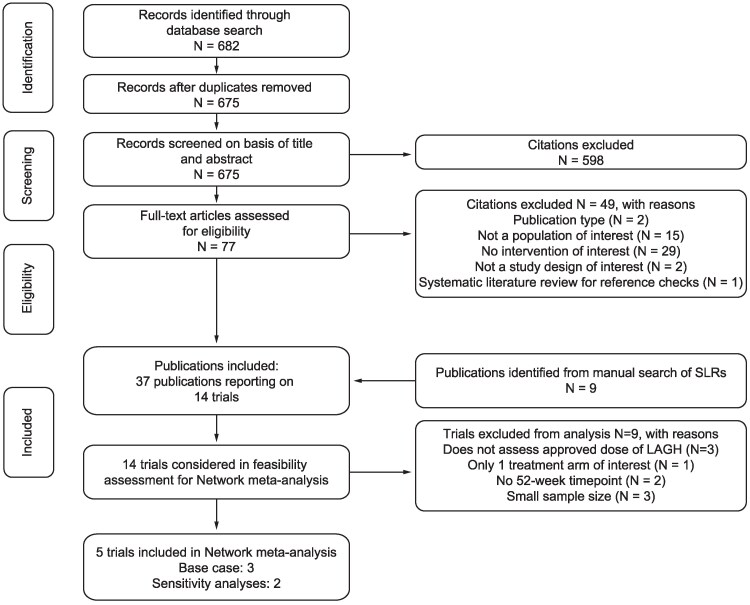
Flow of literature through systematic literature review and feasibility assessment.

Abbreviation: LAGH, long-acting growth hormone.

A total of 675 unique records were identified through database searches and screened. Among these, 77 full-text publications were reviewed. Ultimately, 37 publications that reported on 14 independent trials were included from the database searches and manual checks of bibliographies. Of these 14 trials, 5 were deemed suitable for inclusion in the network meta-analysis [33, 46-49]. Fig. 1 outlines the flow of literature through the systematic literature review and feasibility assessment, and Supplementary Table S2 [41] lists studies excluded from analysis with reasons. Trials must have evaluated an approved dose to be eligible for analysis, as approved doses best reflect clinical practice. Additionally, only trials reporting outcomes at 52 weeks were included in the analysis to reduce heterogeneity caused by variability in shorter-term growth. Notably, the trials of PEGylated GH did not include a 52-week time point and included only 1 treatment arm of interest; thus, no trials assessing this treatment could be included in this analysis [50, 51].

Among the 5 studies deemed feasible to be included in the network meta-analysis, 4 were global, multicenter trials, and the remaining study was conducted in Japan only. All studies were phase 3, open-label, randomized controlled trials, with each including a single LAGH and daily somatropin, and all were sponsored by pharmaceutical companies. Inclusion and exclusion criteria were broadly similar across trials. The sample size ranged from 44 to 228 patients. The LAGH formulations evaluated across trials were LB03002 [48], lonapegsomatropin [33], somapacitan [49], and somatrogon [46, 47]. The daily somatropin treatments included Genotropin [33, 46-48] and Norditropin [49], with doses ranging from 0.175 to 0.24 mg/kg/week (Table 2).

**Table 2.** Study characteristics of the trials included in the network meta-analysis

Study ID NCT No.	Region	Phase	Blinding	Recruitment period	No.	Interventions	Primary inclusion criteria
Thornton et al [33] NCT02781727	International	3	Open-label	2016-2019	162	Lonapegsomatropin 0.24 mg hGH/kg/wk	GHD diagnosis confirmed by 2 GH stimulation tests with peak GH levels ≤10 ng/mL Boys ages 3-12 y; girls ages 3-11 y Tanner stage 1 Height SDS ≤ −2.0 IGF-1 SDS ≤ −1.0 BMI SDS −2 to +2 Bone age ≥6 mo behind chronological age
						Daily somatropin (Genotropin) 0.24 mg/kg/wk	
Deal et al [47] NCT02968004	International	3	Open-label	2017-2019	228	Somatrogon 0.66 mg/kg/wk	GHD diagnosis confirmed by 2 GH stimulation tests with peak GH levels ≤10 ng/mL Boys ages 3-11 y; girls ages 3-10 y Prepubertal Height velocity <25th percentile for chronological age IGF-1 SDS ≤ −1.0 BMI SDS ≥ −2 Bone age < chronological age
						Daily somatropin (Genotropin) 0.24 mg/kg/wk	
Miller et al [49] NCT03811535	International	3	Open-label with blinded height measurements	2019-2021 (end of trial, recruitment end not reported)	200	Somapacitan 0.16 mg/kg/wk	GHD diagnosis confirmed by 2 GH stimulation tests with peak GH levels ≤10.0 ng/mL Boys ages 2.5-11 y; girls ages 2.5-10 y Testes volume <4 mL for males; breast development Tanner stage 1 for females Height SDS ≤ −2.0 Height velocity <25th percentile IGF-1 SDS ≤ −1.0
						Daily somatropin (Norditropin) 0.24 mg/kg/wk	
Horikawa et al [46] NCT03874013	Japan	3	Open-label	NR	44	Somatrogon 0.25, 0.48, and 0.66 mg/kg/wk (each for 2 wk) then 0.66 mg/kg/wk	GHD diagnosis confirmed by 2 GH stimulation tests with peak GH levels ≤6 ng/mL (≤16 ng/mL for GH-releasing peptide-2 provocation test) Boys ages 3-10 y; girls ages 3-9 y Prepubertal Height SDS ≤ −2.0 Height velocity <25th percentile for chronological age IGF-1 SDS ≤ −1.0
						Daily somatropin (Genotropin) 0.175 mg/kg/wk	
Khadilkar et al [48] NCT00271518	International	3	Open-label	NR	180	LB03002 0.5 mg/kg/wk	Idiopathic or organic GHD diagnosis confirmed by 2 GH stimulation tests with peak GH levels ≤7.0 ng/mL Boys ages >3 and <12 y; girls ages >3 and <11 y Prepubertal Height SDS ≤ −2.0 (idiopathic GHD only) IGF-1 SDS ≥0.5 BMI SDS −2 to +2 Bone age ≤ chronological age
						Daily somatropin (Genotropin) 0.21 mg/kg/wk	

Abbreviations: BMI, body mass index; GH, growth hormone; GHD, growth hormone deficiency; hGH, human growth hormone; IGF-1, insulin-like growth factor-1; SDS, SD score.

All trials had a low to medium risk of bias. The main source of bias was the lack of blinding; an open-label design was used in all studies, likely due to the differences in dosing frequency between the LAGH and daily somatropin arms. A figure summarizing the risk of bias for studies contributing to the analysis is presented in Supplementary Fig. S1 [41].

The feasibility assessment identified 3 trials [33, 47, 49] that were similar enough in terms of study designs, comparator treatments, and patient cohorts to be included in the base case analysis (Table 3).

**Table 3.** Baseline characteristics of the studies included in the network meta-analysis

Trial	Deal et al [47]		Thornton et al [33]		Miller et al [49]		Horikawa et al [46]		Khadilkar et al [48]	
**Treatment arm**	Genotropin 0.24 mg/kg/wk	Somatrogon 0.66 mg/kg/wk	Genotropin 0.24 mg/kg/wk	Lonapegsomatropin 0.24 mg/kg/wk	Norditropin 0.24 mg/kg/wk	Somapacitan 0.16 mg/kg/wk	Somatropin 0.175 mg/kg/wk	Somatrogon 0.25, 0.48, and 0.66 mg/kg/wk (each for 2 wks) then 0.66 mg/kg/wk	Genotropin 0.21 mg/kg/wk	LB03002 0.5 mg/kg/wk
**No. assessed**	115	109	56	105	68	132	22	22	87	91
**Male, %**	68.7	75.2	82	82	73.5	75	54.5	40.9	63.2	61.5
**Age, y*^a^***	7.61 (range, 3.05-11.85)	7.83 (range, 3.01-11.96)	8.5 (2.8)	8.5 (2.7)	6.4 (2.4)	6.4 (2.2)	6.78 (2.34)	5.28 (1.84)	7.8 (2.5)	7.8 (2.5)
**Weight, kg*^a^***	NR	NR	21.2 (6.7)	21 (6.5)	16 (4.95)	16.7 (4.6)	NR	NR	17.2 (6.1)	17 (6.1)
**White, %**	74.8	74.3	92.9	95.2	52.9	59.1	NR	NR	NR	NR
**Black, %**	1.7	0	NR	NR	1.5	0	NR	NR	NR	NR
**Asian, %**	18.3	22	NR	NR	41.2	34.8	100	100	NR	NR
**Etiology: Idiopathic, %**	NR	NR	66	65	89.7	87.1	NR	NR	88.5	91.2
**Etiology: organic, %**	NR	NR	16	18	10.3	12.9	NR	NR	11.5	8.8
**Isolated GH deficiency, %**	NR	NR	NR	NR	NR	NR	NR	NR	44.8	45.1
**Multiple pituitary hormone deficiencies, %**	NR	NR	18	17	NR	NR	NR	NR	55.2	54.9
**Bone age, y*^a^***	5.19 (2.45)	5.46 (2.72)	6 (2.7)	5.8 (2.6)	NR	NR	NR	NR	NR	NR
**Bone age-to-chronological age ratio*^a^***	NR	NR	0.7 (0.14)	0.69 (0.16)	NR	NR	NR	NR	0.55 (0.17)	0.54 (0.17)
**Height SDS*^a^***	−2.78 (1.27)	−2.94 (1.29)	−3.00 (0.9)	−2.89 (0.85)	−3.47 (1.52)	−2.99 (1.02)	−2.53 (0.4)	−2.61 (0.44)	−4.36 (1.9)	−4.34 (1.82)
**Height velocity SDS*^a^***	−2.14 (2.02)	−2.20 (2.22)	−3.05 (1.54)	−3.16 (1.57)	−2.52 (1.55)	−2.35 (1.51)	NR	NR	NR	NR
**IGF-1 SDS*^a^***	−1.72 (NR)	−1.95 (NR)	−1.96 (0.98)	−2.08 (0.88)	−2.33 (1.03)	−2.03 (0.97)	−1.62 (0.84)	−1.39 (0.9)	−4.30 (2.04)	−4.23 (2.1)

Abbreviations: GH, growth hormone; IGF-1, insulin-like growth factor-1; NR, not reported; SDS, SD score.

^a^Mean (SD).

**Figure 2.**
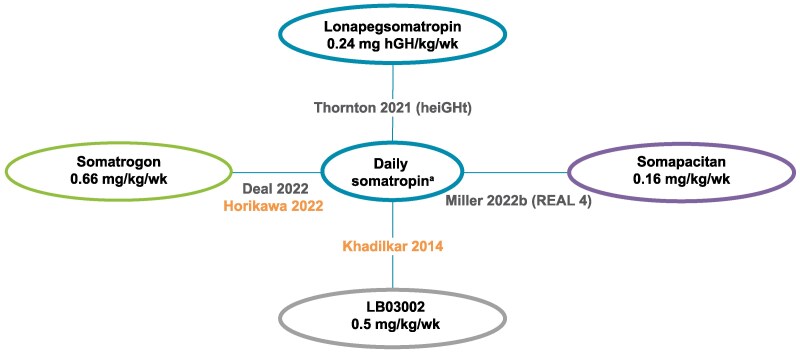
Network of evidence for analysis of LAGHs for pGHD. Base case analysis (gray), included Deal et al [47], Thornton et al [33], and Miller et al [49]. Sensitivity analysis trials (orange) included Horikawa et al [46] and Khadilkar et al [48]. ^a^Dose of daily somatropin was 0.24 mg/kg/week in Thornton et al [33], Deal et al [47], and Miller et al [49]; 0.21 mg/kg/week in Khadilkar et al [48]; and 0.175 mg/kg/week in Horikawa et al [46].

Abbreviations: hGH, human growth hormone; LAGH, long-acting growth hormone; pGHD, pediatric growth hormone deficiency.

The remaining 2 studies, Khadilkar et al [48] and Horikawa et al [46], were included only in the sensitivity analyses due to differences in study design and patient characteristics vs base case trials (Supplementary Table S3) [41]. In short, sensitivity analysis 1 encompassed all trials from the base case analysis and Khadilkar et al [48]. This trial evaluated an intervention exclusively approved and marketed in South Korea in a patient population that differed on key characteristics. The inclusion of this trial yielded a new direct contrast between LB03002 and daily somatropin. Sensitivity analysis 2 included all the trials from the base case analysis along with Horikawa et al [46]. This trial was conducted in Japan and had a notably younger patient population and smaller sample size compared to the studies in the base case analysis. Additionally, the dose of daily somatropin was lower in this study than in the other included trials. The inclusion of Horikawa et al [46] added a second data source for the somatrogon vs daily somatropin treatment contrast. Fig. 2 presents the network diagram illustrating the connection of trials for the base case analysis and sensitivity analyses.

Sufficient data and comparable outcome measures across the included trials allowed for the assessment of relative efficacy and safety between LAGH formulations and to daily somatropin treatment for the following outcomes: annualized height velocity, change from baseline in height SDS, change from baseline in weekly average insulin-like growth factor-1 (IGF-1) SDS, change from baseline in bone age-to-chronological age ratio, and serious adverse events (SAEs) at any time point. Only outcomes data from 52 weeks post-treatment initiation were used in the analyses. Definitions of outcomes analyzed can be seen in Table 4. Supplementary Table S4 [41] outlines the outcomes that were not able to be analyzed with reasons.

**Table 4.** Outcome definitions

Outcome measure used for analysis Analysis output	Deal et al [47]	Thornton et al [33]	Miller et al [49]	Khadilkar et al [48]	Horikawa et al [46]
**Annualized height velocity** Value at 52 wk Mean difference	[(mo 12 height – baseline height)/(mo 12 date – baseline date)] × 365.25	[change in height (cm)/change in time (d)] × 365 d	(height at × wk visit—height at baseline)/(time from baseline to × wk visit in y)	Calculated from linear regression of height against time, using all available height measurements from previous 12 mo	[(month 12 height – baseline height)/(mo 12 date – baseline date)] × 365.25
**Height SDS: standard** Change from baseline to 52 wk Mean difference	2000 CDC Growth Charts	CDC 2000/Kuczmarski method	CDC standards	CDC 2000 reference data	Age and sex standards listed in national survey in y 2000 MHLW Infant and Children's Growth Survey Report (ages 0-6 y) MEXT Statistics Report (ages 6-17 y)
**IGF-1: standard** Change from baseline to 52 wk Mean difference	Age and sex standards listed in 2014 *JCEM* article by Bidlingmaier et al [52]	Age and sex standards listed in 2014 *JCEM* article by Bidlingmaier et al [52]	Unclear	Age-specific data from normal population in article by Elmlinger et al [53]	Age and sex standards listed in article by Isojima et al [54]
**IGF-1: measurement**	A previously developed indirect response pharmacokinetic/pharmacodynamic model (Fisher et al [55]) was applied to IGF-1 observations to estimate IGF-1 SDS profiles over dosing interval	Estimated average based on nonlinear mixed-effect population pharmacodynamic modeling	Model-derived weekly average	Blood samples for IGF-1 measurement for LB03002 group taken d 4 post dose to best approximate mean IGF-1 SDS over 1-wk dosing interval per Fisher et al [55] Blood samples taken ∼12 h post dose in daily somatropin group	Blood samples for IGF-1 measurement for somatrogon group taken d 4 post dose to best approximate mean IGF-1 SDS over 1-wk dosing interval per Fisher et al [55]
**Bone age-to-chronological age ratio** Change from baseline to 52 wk Mean difference	Bone age by Greulich-Pyle method divided by chronological age Bone age decimal value: y + mo/12 Chronological age: age in y + (assessment date – date of most recent birthday)/365.25	Ratio of bone age-to-chronological age advancement	Analyzed using analysis of covariance model on change in bone age-to-chronological age ratio assessed at wk 52	Bone age by Greulich-Pyle method	Bone age by Tanner-Whitehouse 2 method
**SAEs** Proportion of patients experiencing any event by 52 wk OR	Individuals with SAEs	SAEs unrelated to study drug (effectively total SAEs as no patients experienced SAEs related to study drug)	Serious events	SAEs	NR

Results for mean differences are statistically significant if the 95% CrI does not include 0; results for ORs are statistically significant if the 95% CrI does not include 1.

Abbreviations: CDC, US Centers for Disease Control and Prevention; CrI, credible interval; *JCEM*, Journal of Clinical Endocrinology and Metabolism; IGF-1, insulin-like growth factor-1; MEXT, Ministry of Education, Culture, Sports, Science and Technology, Japan; MHLW, Ministry of Health, Labour, and Welfare of Japan; NR, not reported; OR, odds ratio; SAE, serious adverse event; SDS, SD score.

### Network Meta-Analysis Results

#### Base case analysis

**Figure 3.**
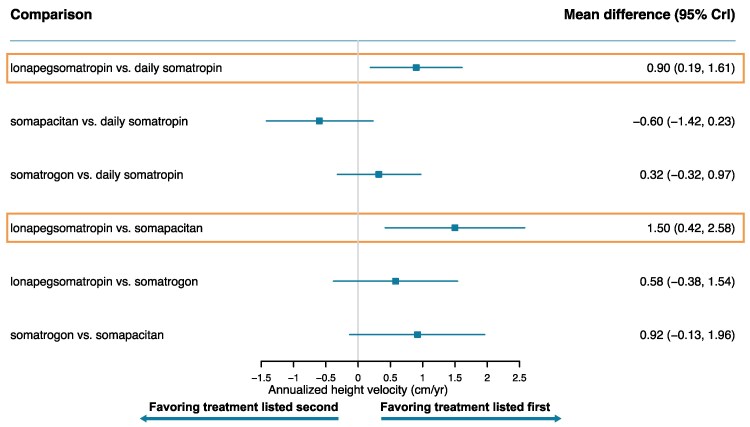
Forest plot of annualized height velocity at 52 weeks—base case. Orange box indicates significant results.

Abbreviation: CrI, credible interval.

**Figure 4.**
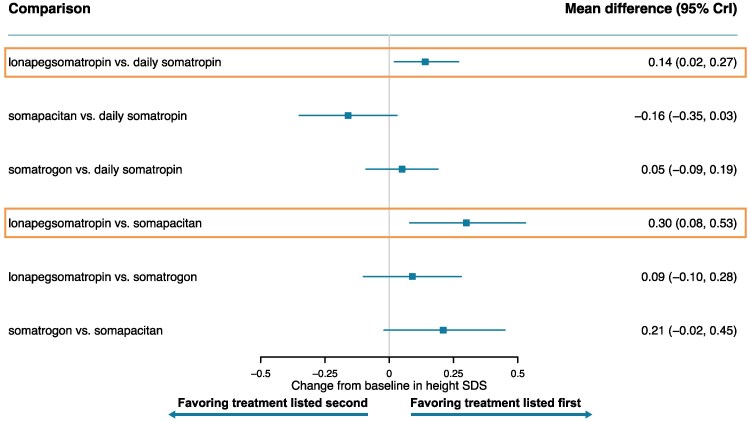
Forest plot of height SDS change from baseline—base case. Orange box indicates significant results.

Abbreviations: CrI, credible interval; SDS, SD score.

**Figure 5.**
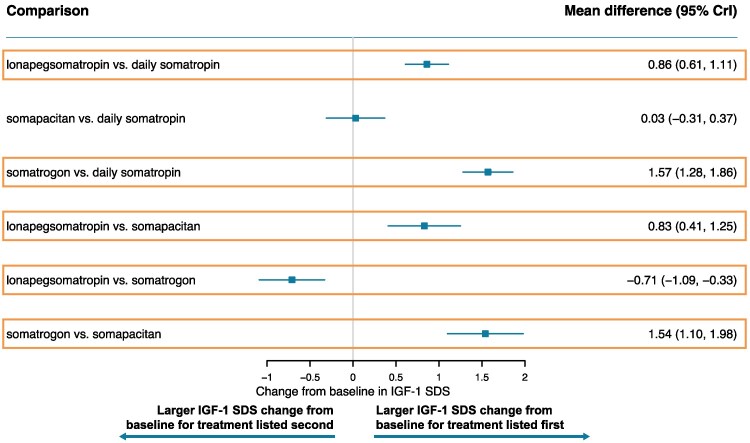
Forest plot of IGF-1 SDS change from baseline—base case. Orange box indicates significant results.

Abbreviations: CrI, credible interval; IGF-1, insulin-like growth factor-1; SDS, SD score.

**Figure 6.**
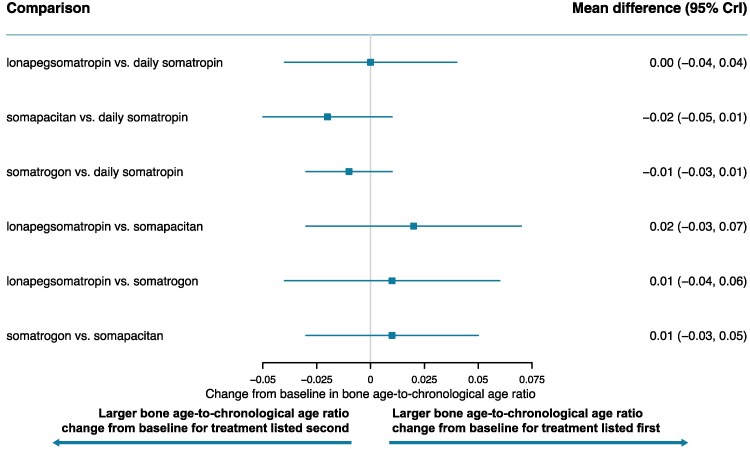
Forest plot of bone age-to-chronological age ratio change from baseline—base case. Orange box indicates significant results.

Abbreviation: CrI, credible interval.

**Figure 7.**
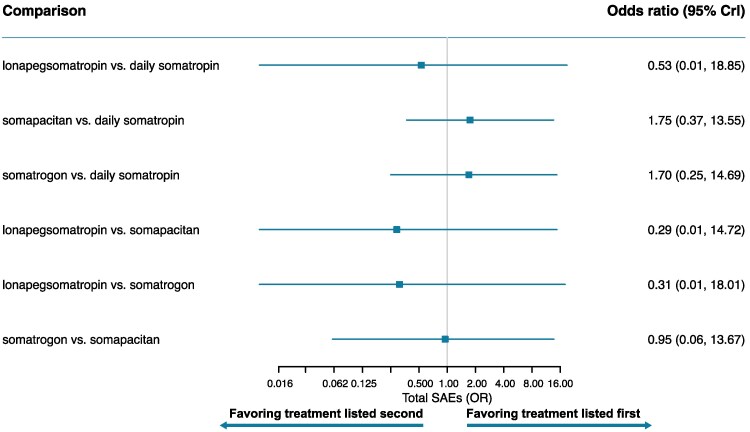
Forest plot of total SAEs—base case. Orange box indicates significant results.

Abbreviations: CrI, credible interval; OR, odds ratio; SAE, serious adverse event.

Figs. 3 to 7 display the results of the base case analysis for all outcomes. Lonapegsomatropin had a statistically significantly higher annualized height velocity (cm/year) at week 52 than both somapacitan (mean difference: 1.50 cm/year [95% CrI, 0.42-2.58]) and daily somatropin (mean difference: 0.90 cm/year [95% CrI, 0.19-1.61]). No other comparisons showed significant differences for this outcome. Surface under the cumulative ranking curve values indicated that lonapegsomatropin had the highest probability (88%) of achieving the highest annualized height velocity at week 52. Surface under the cumulative ranking curve results for all outcomes are available in Supplementary Tables S5 to S9 [41].

Lonapegsomatropin also showed a significantly greater increase from baseline in height SDS than both somapacitan (mean difference: 0.30 [95% CrI, 0.08-0.53]) and daily somatropin (mean difference: 0.14 [95% CrI, 0.02-0.27]). No statistically significant differences were found between other treatments for this outcome. The surface under the cumulative ranking curve values indicated that lonapegsomatropin had the highest probability (82%) of achieving the largest increase from baseline in height SDS.

Somatrogon induced a significantly greater change from baseline in average IGF-1 SDS than all other comparators: somapacitan (mean difference: 1.54 [95% CrI, 1.10-1.98]), lonapegsomatropin (mean difference: 0.71 [95% CrI, 0.33-1.09]), and daily somatropin (mean difference: 1.57 [95% CrI, 1.28-1.86]). Lonapegsomatropin showed a significantly greater change from baseline in average IGF-1 SDS than somapacitan (mean difference: 0.83 [95% CrI, 0.41-1.25]) and daily somatropin (mean difference: 0.86 [95% CrI, 0.61-1.11). Notably, the mean weekly average IGF-1 SDS values for all treatments remained within the normal range (−2 to +2) at week 52. The surface under the cumulative ranking curve values indicated that somatrogon had the highest probability of achieving the largest increase from baseline in average IGF-1 SDS (100%).

There were no significant differences observed in the change from baseline in bone age-to-chronological age ratio or total SAEs.

#### Sensitivity analyses

Sensitivity analysis 1 added a direct comparison between LB03002 and daily somatropin; sensitivity analysis 2 provided an additional data source for the direct comparison of somatrogon to daily somatropin, although the dose of daily somatropin was notably lower than that used in the other trials. In sensitivity analysis 1, compared with LB03002, lonapegsomatropin had a significantly higher follow-up annualized height velocity at week 52 and somatrogon had a significantly higher change from baseline in average IGF-1 SDS. LB03002 showed a significantly higher change from baseline in bone age-to-chronological age ratio than somapacitan. There was no change in direction or significance of any of the comparisons included in the base case. In sensitivity analysis 2, the comparisons for somatrogon vs somapacitan and somatrogon vs daily somatropin showed a significantly higher annualized height velocity at 52 weeks and a significantly larger change from baseline in height SDS, whereas in the base case analysis, these comparisons showed only numerical differences. This addition did not affect other efficacy or safety comparisons. Full results from sensitivity analyses are presented in Supplementary Figs. S2 to S11 and in Supplementary Tables S10 to S19 [41].

## Discussion

With the recent emergence, approval, and commercialization of several LAGH products for pGHD, the portfolio of treatment options has expanded from only daily somatropin. In addition to bringing a new dosing regimen to GH treatment, the development of LAGHs has also introduced molecular diversity to pGHD treatments. LAGHs differ in their molecular structure and the moieties used to make the LAGH result in pharmacokinetic and pharmacodynamic differences. This may affect efficacy and safety as a result of altered tissue distribution [21], but no trial has directly compared the efficacy and safety of the various LAGHs.

The objective of this study was to provide a systematic literature review– and Bayesian network meta-analysis–based comparison of the efficacy and safety of the various LAGHs. While there are 3 recent publications quantitatively synthesizing the literature on LAGHs in pGHD [36, 38, 39], there are key differences between these publications and the present analysis. One study conducted pairwise meta-analyses and thus did not generate comparisons of the LAGHs to each other [36]. Another Bayesian network meta-analysis combined multiple doses into one treatment node. This is not reflective of clinical practice and may introduce heterogeneity by assuming that the relative effects of a given LAGH and daily somatropin are the same regardless of dose. Additionally, in that analysis, the authors compared studies reporting outcomes at 26 weeks and at 52 weeks, which introduces heterogeneity due to the variations in growth patterns over the first year after initiating GH treatment [39]. Another recent network meta-analysis used a frequentist approach for the analysis [38], while our analysis used a Bayesian approach, which allowed for treatment rankings, enhancing the interpretation of network meta-analysis results, and included 2 additional outcomes. However, the results are similar between the 2 analyses.

While all treatments performed similarly on the only analyzable safety outcome, SAEs, significant differences were seen between LAGHs in efficacy outcomes. For both annualized height velocity and change from baseline in height SDS, lonapegsomatropin was associated with statistically significantly larger improvements when compared with somapacitan and somatropin, and no other comparisons led to significant differences. Lonapegsomatropin also showed the highest probability of all comparators in the network meta-analysis of leading to the largest annualized height velocity and increase from baseline in height SDS at 52 weeks.

There were differences in the way IGF-1 SDS was reported as a binary outcome (eg, the percentage of children with pGHD experiencing IGF-1 SDS >2), which prevented the joining of all trials in a network meta-analysis for this outcome. Variations in the definitions included the cutoff (>2 or ≥2), the number of measurements required (at any time during the study or 2 consecutive measurements), and whether the measurement used was an average IGF-1 SDS. However, all trials used an average IGF-1 SDS (referring to IGF-1 drawn at a time point corresponding to the mean IGF-1 exposure for the week) when reporting the change from baseline, allowing for a network meta-analysis of this outcome. This analysis used trials reporting mean or average IGF-1 SDS [33, 49] and those reporting modeled mean IGF-1 SDS [33, 47, 48]. In all included trials in this network meta-analysis [33, 46-49], there were downtitrations of LAGH doses for IGF-1 levels exceeding the normal range in a small proportion of participants. Nevertheless, the mean values for average weekly IGF-1 SDS reported in the included studies were all within the normal range.

Another concern of GH therapy is the possibility of untoward bone-age progression that may result in premature closure of growth plates, leading to premature cessation of growth [10]. The present analysis showed that there were no significant differences in the effects of LAGHs and daily somatropin on bone age-to-chronological age ratio. Actual change from baseline in bone age-to-chronological age ratio was small in all trials (range, 0.05-0.08 in the base case trials; 0.04-0.15, including sensitivity analyses trials).

A key limitation of this analysis is the lack of analyzable safety outcomes. Of the 8 safety outcomes considered, only 1 (SAEs) was able to be analyzed. Total adverse events (AEs) were unable to be analyzed due to differing definitions across the included trials. High AE rates reported by the trials (range, 71.2%-100% for LAGH arms vs 60.3%-86.4% for daily somatropin arms) may be partially driven by the percentage of patients experiencing a painful injection-site reaction (range, 1.2%-43.2% for all patients). However, the recording of injection-site pain reactions and overlap with total AEs was inconsistent among studies. Thus, neither total AEs nor injection-site reactions could be determined to be comparable across trials. Non neutralizing anti-hGH antibodies and dose reductions also had different definitions across trials, and neutralizing anti-hGH antibodies and discontinuations due to AEs could not be analyzed due to the low number of events across all trials. The general safety profile of all GH treatments is further supported by the low rates of SAEs reported in the included trials (1.0%-9.1% for LAGH arms vs 1.7%-9.1% for daily somatropin arms).

The small network available precluded any adjustment for differences in baseline characteristics; sensitivity analyses were the only possible method for exploring the effects of these types of differences. The lower dose of daily somatropin in Horikawa et al [46] may have amplified the perceived relative effectiveness of the LAGH used in that trial (somatrogon). While there were also some differences in baseline characteristics, results of the sensitivity analyses were largely consistent with those of the base case. A matching-adjusted indirect comparison would be an alternative to a network meta-analysis that would allow for adjustment for patient baseline characteristics. However, separate matching-adjusted indirect comparisons must be conducted for each comparison, and individual patient data are required for at least one trial in each comparison, which raises the barrier to conducting such analyses. Because of the size and shape of the available network, formal estimates of heterogeneity and consistency were not possible, and analyses were limited to fixed-effects models.

Limited reporting in the identified literature of specific data elements required some estimation and assumptions to create an analyzable data set. Some of the means and SDs in this analysis are estimated by figure extraction; the quality of these estimations varies based on the quality and accuracy of the source figure. The lack of reporting of SDs (or measures through which they could be calculated) for baseline and follow-up raw values or changes from baseline meant that these were often imputed. When estimating change from baseline SD using baseline and follow-up SDs, a correlation term (*r*) is required. There were insufficient data to estimate *r* per outcome type, so it was estimated based on the complete pairs (n = 16) of baseline/follow-up values across all outcomes. The imputation of change from baseline SDs was based on some values that were estimated from imputed baseline and/or follow-up raw values, thereby compounding uncertainty.

One of the key strengths of this analysis was the focus on approved doses of LAGHs. Early-phase dose-finding trials often include doses that are ultimately found to be ineffective. While there is often a difference in the performance of treatments in clinical trials and the real world, using only approved doses ensures that the assessed doses are the most clinically effective and allows this analysis to be more relevant to clinical practice. Additionally, we used only data reported at 52 weeks. By using one time point for all trials, we eliminated a key source of heterogeneity due to variations in growth patterns and, by using the latest time point in the double-blind phase of the trials, we were able to focus on longer-term effects. The Bayesian framework used for the network meta-analysis allows the calculation of surface under the cumulative ranking curve scores to aid in the interpretation of the results.

### Conclusions

We compared the efficacy and safety of different approved LAGH formulations, including a comparison with daily somatropin therapy, using a Bayesian approach. In this network meta-analysis, lonapegsomatropin showed statistically significantly higher 52-week annualized height velocity and a larger change from baseline in height SDS than somapacitan and daily somatropin. Somatrogon showed a larger change from baseline in average IGF-1 SDS than somapacitan and lonapegsomatropin. However, the superiority of somatrogon in eliciting an IGF-1 SDS response did not translate into a statistically superior annualized height velocity at 52 weeks vs any comparator. The LAGH formulations analyzed in the base case use different mechanisms to increase half-life and allow for once-weekly dosing, where some are permanently altered GH derivatives (somatrogon and somapacitan) and others release unmodified GH (lonapegsomatropin) to engage the GHR [56]. The effect of molecular differences in LAGH formulations on the efficacy outcomes analyzed in this study and on other effects of GH, such as metabolic health, cannot be determined by a network meta-analysis. The full picture of the effects of GH and the differences in how LAGHs bring about such effects should be considered when determining the best treatment option for individuals with pGHD.

## Data Availability

Some or all data sets generated during and/or analyzed during the present study are not publicly available but are available from the corresponding author on reasonable request.

## Abbreviations

AEs: adverse events
BMI: body mass index
CI: confidence interval
CrI: credible interval
GH: growth hormone
GHD: growth hormone deficiency
GHR: growth hormone receptor
hGH: human growth hormone
IGF-1: insulin-like growth factor-1
kDa: kilodalton
LAGH: long-acting growth hormone
NICE: National Institute for Health and Care Excellence
pGHD: pediatric growth hormone deficiency
rhGH: recombinant human growth hormone
SAEs: serious adverse events
SDS: SD score
